# Supplementary material for: UK consultants’ experiences of the decision-making process around referral to intensive care: an interview study
Source: BMJ Open. 2021 Mar 24;11(3):e044752. doi: 10.1136/bmjopen-2020-044752 (PMC7993217; doi:10.1136/bmjopen-2020-044752)
Supplement: Supplementary data [file bmjopen-2020-044752supp001.pdf]

REC number: 15/WM/0025

Understanding and improving the decision-making process for treating people who are critically ill

Non referring clinicians

1. I am just curious to know how come you volunteered for this interview?

2. In the past three weeks have you referred to ICU any patients under your care?

   A) **If yes,** starting with the most recent patient, can you talk me through how you made the decision to contact the critical care team (repeat for up to 5 cases)
   Follow-up: Who did you contact and for what reason? What do you think influenced you when making this decision? Circumstances? Persons? What was the outcome, admission to ICU or not? What do you think was the most important to take into account when you made the decision?

   B) **If no**, did you consider referring patients, but made the decision not to? Please, tell me about how you reasoned about not referring?
   Follow-up: *What do you think influenced you when making this decision? Circumstances? Persons? What do you think was the most important to take into account when you made the decision?*

   C) **If you have not considered referral**, can you talk me through reasons for not referring to ICU?

3. What is your general experience of the decision-making referring patients to ICU? *Follow-up: How do you think your way of making decisions to refer compare with your colleagues?*

4. Thinking more generally, how could decision making whether to admit patient or not to ICU be improved?

5. What type of support would be useful when facing this type of decision? *Follow-up: What format of this support would be useful?*

6. Are you aware of any policies about this kind of decision-making?

Background
Age:
Specialty:
Years as consultant:
Graduation year:

IS3 clinician interviews WP1 V6.1 23.10.15
