# Supplementary material for: UK consultants’ experiences of the decision-making process around referral to intensive care: an interview study
Source: BMJ Open. 2021 Mar 24;11(3):e044752. doi: 10.1136/bmjopen-2020-044752 (PMC7993217; doi:10.1136/bmjopen-2020-044752)
Supplement: Supplementary data [file bmjopen-2020-044752supp002.pdf]

**Themes**	**Subthemes**	**Quotes**
Burdensome doubt	Being affected by the gravity of critical illness	*…and just conveyed to them (intensive care team) of how unwell this patient was and if there was a window of opportunity that it was only going to be a very small one.* (Acute and Emergency Medicine consultant, Hospital 1)<br><br>*It's a life or death situation that needs to have a thorough discussion.* (Renal and Emergency Medicine consultant, Hospital 6)<br><br>*It's that reassurance that it has been taken seriously and it's not a light decision. People have perhaps thought about you know the alternative to intensive care. So I think it's very easy to say, "Well this person's not for intensive care," without thinking what, what does that mean for the patient? What are you going to do instead?* (Internal Medicine consultant, Hospital 2)
	Pondering about what is meaningful for the patient	*The chances of there being a worsening function are high. So very often we might get somebody to survive but they won't walk.* (Geriatric consultant, Hospital 2)<br><br>*But I don't think ultimately he would benefit from it, I think the burden of it would probably outweigh any likelihood and the best you could hope for would be to get him back to his pre-morbid condition which was happy and smiley, interacting a little but completely physically dependent. So I'm not sure that would be a helpful thing for him.* (Internal Medicine consultant, Hospital 2)<br><br>*When I feel that it wouldn't be beneficial for them to go to intensive care and that's sometimes the patients that are very frail or have a lot of comorbidities who in my experience of intensive care, my interaction with them, I don't think would be successful, I don't think ICU would change their end kind of outcome, where I feel we would be doing more harm to them that the benefit.* (Acute and Emergency Medicine consultant, Hospital 1)
	Ambivalence in searching for the patient's voice	*Well again I think we try and involve the family but often again although they're in a slightly better position in that they're not ill when they're trying to weigh up decisions, it's still difficult to grasp all the information and the implications of it.* (Renal and Emergency Medicine consultant, Hospital 5)<br><br>*A patient who's clearly just reaching the end of their life, becomes desperate and wants you to try everything. … we're inviting the patients to give their opinion but then we're going to ignore it if we think it's the wrong thing,*

		that's very difficult for medicine to deal with them. I don't know how we should approach that really. (Cardiology consultant, Hospital 5)<br><br>*Not to lose sight of what we think is best for the patient and to make sure that we stick to the factors that present themselves and that we make level headed decisions for the patients and I think we are very aware that patients may have got an overly optimistic view of what ICU can achieve.* (Oncology consultant, Hospital 2)
Emerging joint understanding	Comprehension by seeing the patient at the bedside	*There are some patients as I say in that grey area that I feel very much it's eyeballing the patient and getting a feel for them as to what that decision should be and in those patients I think that your personal review is important. Just like when I make a decision as to whether somebodies going to go to theatre or not.* (Colorectal surgeon, Hospital 5)<br><br>*If they're starting to get tired, they look tired, you can sometimes by looking at them sort of, sometimes it's a gut feeling. I know that is difficult to put in a guideline isn't it?* (Neurology consultant, Hospital 6)<br><br>*You find a very different picture and the patient's much weller than you expected them to be or much sicker than you expected them to be. Y it's hard to say, "No," when you've seen the patient, you have to justify that and so you have to be more of a doctor.* (Acute and Emergency Medicine consultant, Hospital 4)
	Requiring senior experience to grasp clinical complexity	*Sometimes things may happen too acutely and maybe out of hours for that initial consultant to consultant discussion to happen but I think in a, in a sort of fairly short time frame there ought really to be a consultant and consultant discussion about the patient so that both sides can fully understand the context.* (Haematology consultant, Hospital 3)<br><br>*At the end of the day there's going to be no substitute for a trained clinician, who's experienced to handle these quite complex sort of pictures and to guide through those sort of decisions.* (Infectious Disease consultant Hospital 5)<br><br>*Trying to do it in a thoughtful senior clinician based manner rather than it's 3 in morning what should we do?* (Internal Medicine consultant, Hospital 2)
	Needing engagement for broadened perspectives	*Who (intensive care team) are extremely helpful and are always happy to listen and always happy to come and see the patient and have a positive, you know, constructive discussion.* (Oncology consultant, Hospital 2)

Heidenreich K, *et al. BMJ Open* 2022; 11:e044752. doi: 10.1136/bmjopen-2020-044752

		They're coming from a different angle, I think usually if you phrase this on not sure what the best solution is here because of x, y, z and then talk that through that does tend to be the better sort of collaborative sort of approach. (Acute and Emergency Medicine consultant, Hospital 1)<br><br>*There are situations where I'm very confident that I know with that clinical situation well enough to be able to make very clear decisions, I've got no hesitation of doing that. But there are situations where there is genuine doubt and the view would be at that stage having somebody else from a different, coming from a different angle, who's very helpful to see and if critical care felt that they were able to do something to help them then that would be fine* (Hepatology consultant, Hospital 2)
Responding to mutual moral duties	Meeting in person to acknowledge concern	*It's just different, you can do it on the phone but you don't get the same, on a phone it's very hard to relay all that information, we've all been on call and at the end of the phone and you just don't get a picture of what's going on, whereas when you talk to somebody you ask different questions, you have a much, it's a more informal discussion I think when it's face to face.* (Colorectal surgeon, Hospital 5)<br><br>*So I think that, that communication is absolutely crucial and sometimes you know there's something that you may not have thought about but, you actually know the answer to and, rather than this going backwards and forwards if you have either a direct discussion or you're present at the time the discussion is being had, I find that very helpful.* (Oncology consultant, Hospital 2)<br><br>*So it's about a conversation between 2 clinicians and I, I honestly still believe the best, that's best on face to face where ever possible, it's not always possible but where ever possible.* (Colorectal surgeon, Hospital 5)
	Protecting the patient through advocacy	*When I'm referring a patient to ITU, I am acting as that patient's advocate and THAT patient's advocate only and the capacity is somebody else's problem. By heck! That is my patient and I want the best for that patient!* (Respiratory physician consultant, Hospital 6)<br><br>*I see myself as the advocate of the patient because he's on my ward, so I stand for him and ITU needs to assess and then needs to convince me why not. That is really my take. Because I would not ask if I don't want the patient to lie in intensive care.* (Renal and Emergency Medicine consultant, Hospital 6)<br><br>*Whether you have a bed or not, I'm not asking you if you've got a bed, I'm asking you for your opinion as an intensivist whether you think this patient's condition would best be managed on an intensive care unit. Once we've decided that*

Heidenreich K, *et al. BMJ Open* 2022; 11:e044752. doi: 10.1136/bmjopen-2020-044752

		is the best place we can then decide where we can find an intensive care bed or how we could facilitate a bed being available. (Renal Medicine consultant, Hospital 3)
	Building relationships for confidence	*If they know you and they trust you and they know what, what you usually do or whether you look after your patients and how you work, most of you will then take that on board and say, "Well fine…" (*Colorectal surgeon, Hospital 5)<br><br>*If I go to them and say, "Look this is the difficulty I have," I've always found, I've always found everybody to be quite reasonable and try to accommodate and weighing up the risks and benefits of the patient. I've never found an ITU consultant to be defensive and saying they don't want to take a patient or to block a patient off, that's not really been the case. If you can, if they can understand why you're doing something and what the surgery's about but that's also I think… the factor of them whether they learn that what you do is reasonable or not. I think it depends on your relationship with them and some cases I think perhaps there will be a lesser understanding for why somebody wants a patient on ITU if historically that decision making has not always been good form the surgeon's side.* (Orthopedic surgeon, Hospital 2)<br><br>*…they know that we don't ask for flippant requests and that if we want help, we need it and so I think that they're extremely supportive.* (Haematology consultant, Hospital 1)

Heidenreich K, *et al. BMJ Open* 2022; 11:e044752. doi: 10.1136/bmjopen-2020-044752
