# Supplementary material for: UK consultants’ experiences of the decision-making process around referral to intensive care: an interview study
Source: BMJ Open. 2021 Mar 24;11(3):e044752. doi: 10.1136/bmjopen-2020-044752 (PMC7993217; doi:10.1136/bmjopen-2020-044752)
Supplement: Author's manuscript [file bmjopen-2020-044752.draft_revisions.pdf]

BMJ Open is committed to open peer review. As part of this commitment we make the peer review history of every article we publish publicly available.

When an article is published we post the peer reviewers' comments and the authors' responses online. We also post the versions of the paper that were used during peer review. These are the versions that the peer review comments apply to.

The versions of the paper that follow are the versions that were submitted during the peer review process. They are not the versions of record or the final published versions. They should not be cited or distributed as the published version of this manuscript.

BMJ Open is an open access journal and the full, final, typeset and author-corrected version of record of the manuscript is available on our site with no access controls, subscription charges or pay-per-view fees (http://bmjopen.bmj.com).

If you have any questions on BMJ Open's open peer review process please email info.bmjopen@bmj.com

BMJ Open

Referring patients to intensive care: consultant doctors' experiences of the decision-making process

Journal:	*BMJ Open*
Manuscript ID	bmjopen-2020-044752
Article Type:	Original research
Date Submitted by the Author:	15-Sep-2020
Complete List of Authors:	Heidenreich, Kaja; University Health Care Research Center, Faculty of Medicine and Health;<br>Slowther, Anne-Marie; University of Warwick, Warwick Medical School<br>Griffiths, Frances; University of Warwick, Warwick Medical School<br>Bremer, Anders; Linnaeus University, Faculty of Health and Life Sciences, Department of Health and Caring Sciences<br>Svantesson, Mia; Orebro University, Centre for Healtcare Sciences Faculty of Health and Medicine
Keywords:	MEDICAL ETHICS, QUALITATIVE RESEARCH, Adult intensive & critical care < INTENSIVE & CRITICAL CARE

SCHOLARONE™
Manuscripts

BMJ

I, the Submitting Author has the right to grant and does grant on behalf of all authors of the Work (as defined in the below author licence), an exclusive licence and/or a non-exclusive licence for contributions from authors who are: i) UK Crown employees; ii) where BMJ has agreed a CC-BY licence shall apply, and/or iii) in accordance with the terms applicable for US Federal Government officers or employees acting as part of their official duties; on a worldwide, perpetual, irrevocable, royalty-free basis to BMJ Publishing Group Ltd ("BMJ") its licensees and where the relevant Journal is co-owned by BMJ to the co-owners of the Journal, to publish the Work in this journal and any other BMJ products and to exploit all rights, as set out in our licence.

The Submitting Author accepts and understands that any supply made under these terms is made by BMJ to the Submitting Author unless you are acting as an employee on behalf of your employer or a postgraduate student of an affiliated institution which is paying any applicable article publishing charge ("APC") for Open Access articles. Where the Submitting Author wishes to make the Work available on an Open Access basis (and intends to pay the relevant APC), the terms of reuse of such Open Access shall be governed by a Creative Commons licence – details of these licences and which Creative Commons licence will apply to this Work are set out in our licence referred to above.

Other than as permitted in any relevant BMJ Author's Self Archiving Policies, I confirm this Work has not been accepted for publication elsewhere, is not being considered for publication elsewhere and does not duplicate material already published. I confirm all authors consent to publication of this Work and authorise the granting of this licence.

For peer review only

Referring patients to intensive care: consultant doctors' experiences of the decision-making process

Kaja Heidenreich [1]

Anne Slowther [2]

Frances Griffiths [2]

Anders Bremer [3]

Mia Svantesson[1]

1) University Health Care Research Centre, Faculty of Medicine and Health, Örebro University, Örebro, Sweden
2) Division of Health Sciences, Warwick Medical School, University of Warwick, UK
3) Faculty of Health and Life Sciences, Linnaeus University, Växjö, Sweden

Corresponding author:

Kaja Heidenreich

University Health Care Research Centre
S-huset vån 2
SE-701 85 Örebro
Sweden

Telephone +46 70 269 4705

E-mail: kaja.heidenreich@regionorebrolan.se

Kaja Heidenreich: PhD Student, University Health Care Research Center, Faculty of Medicine and Health, Örebro University, Sweden
Anne Slowther: Professor of Clinical Ethics, Warwick Medical School, University of Warwick, UK
Frances Griffiths: Professor of Medicine in Society, Warwick Medical School, University of Warwick, UK
Anders Bremer: Senior Lecturer, Associate professor, Faculty of Health and Life Sciences, Linnaeus University, Växjö, Sweden
Mia Svantesson: Senior Research Fellow, Associate professor, University Health Care Research Center, Faculty of Medicine and Health, Örebro University, Sweden

Word count: 5175 words

Key words: Critical care, Clinical Decision-making, Clinical Ethics, Ethical Theory, Physicians.

For peer review only
For peer review only - http://bmjopen.bmj.com/site/about/guidelines.xhtml

ABSTRACT

Objectives: The decision whether to initiate intensive care for the critically ill patient involves ethical questions regarding what is good and right for the patient. It is not clear how referring doctors negotiate these issues in practice. The aim of this study was to illuminate the meaning of consultants' lived experiences of the decision-making process when referring patients to intensive care.

Design: Qualitative interviews were analysed according to a phenomenological hermeneutical method.

Setting and participants: Consultant doctors (n=27) from departments regularly referring patients to intensive care in six UK hospitals.

Results: In the precarious situation of critical illness, trust in the decision-making process is needed and can be nurtured through the way in which the process unfolds. When the clinical scenario of a critically ill patient does not have obvious right or wrong answers as to what ought to be done, how the decision is made and how the process unfolds is morally important. Through acknowledging the burdensome doubts in the process, contributing to an emerging understanding of the patient's situation, and responding to the moral duties of the doctors involved, trust can be nurtured and moral responsibility assumed.

Conclusions: A trusted decision-making process is engendered by a responsible proximity between the stake-holding doctors to assume and support their moral responsibility for the further care of the critically ill patient.

Strengths and limitations of this study

- The phenomenological hermeneutic method enabled the meanings of the lived experiences of a morally complex decision-making process to be captured, resulting in a deeper understanding of this phenomenon.
- The sample included hospitals of different sizes and a range of clinical specialties, which provided a variety of experiences to inform the study question.
- The sample size was large for a phenomenological hermeneutical analysis, which challenged the depth of analysis.

For peer review only - http://bmjopen.bmj.com/site/about/guidelines.xhtml

BACKGROUND

Admission to an intensive care unit (ICU) allows critically ill patients to access life-saving treatment. However, the short- and long-term mortality of ICU patients is substantial (1-3). In the UK, 20% of patients admitted to the ICU die in the unit and, additionally, 10% will die before hospital discharge (4). The care received involves invasive, distressing and potentially harmful interventions, as well as psychological stress for both patients and their families (5-8). Patients' lived experiences, reported after a stay in the ICU, have revealed strange and frightening memories as well as a process of struggling to find meaning in their ongoing lives (9, 10).

Given the burden of intensive care therapy and the limited prognosis, intensive care treatment will not be beneficial for all patients, and ward-based care or palliative care might be more beneficial (11). The decision whether to refer and admit a patient to ICU is often complex, time pressured, and made in the context of clinical uncertainty with limited knowledge of the patient's wishes for their further care (11). This complex situation was the departure for the project, "Understanding and improving the decision-making process surrounding admission to the intensive care unit", conducted at six NHS trusts in the Midlands, UK (11). The present paper is a sub-study of the wider project.

Previous research suggests that there is large variation in how decisions about admission to ICU are made, depending on the individual clinician, unit, hospital and country characteristics (12-15). The Society of Critical Care Medicine offer guidelines for admission, discharge and triage, but only sparse guidance about the associated ethical issues and the complex decision-making processes, leaving multiple unanswered research questions (16). Previous research has focused on justice and resource allocation (17-21) and factors that influence the decisions (22, 23) and has been considered only from the perspective of intensivists (24, 25).

The main project used a focussed ethnography method deploying participant observation and formal interviews to describe and understand the decision-making process (11). The project included the perspective of referring doctors from wards and emergency departments, as little is known about their experiences of the decision-making process. In addition, clinical experience is valuable in developing deeper knowledge about these complex issues and, therefore, the perspective of consultant doctors was sought to develop further understanding of the process.

Thus, the aim of the present sub-study was to illuminate the meaning of consultant doctors' lived experiences of their decision-making processes when referring patients to intensive care.

For peer review only - http://bmjopen.bmj.com/site/about/guidelines.xhtml

METHOD

Design

The present paper is an analysis of a sub-set of the data collected in the ethnographic study; interviews with consultants who regularly refer patients to ICU. We analysed the interview data using phenomenological hermeneutics as our analysis method (26).

The phenomenological hermeneutical method was developed for the interpretation of lived experiences of ethically difficult situations, so-called "lived ethics" (26). The epistemological basis of this approach is that "lived ethics", i.e., how people act and think about real moral situations, are not easily accessible in empirical research because most people have profound difficulties when explaining their moral actions. The method was therefore developed to reveal the underlying ethics through interpreting the meaning of the lived experiences of interviewees based on their narratives about ethical difficulties (26). The phenomenological element aims to describe and understand the lived experiences of "actions, attitudes, relations or other human matters as ethically good or bad" and to "understand this good as the essential meaning of ethically good phenomena (or the essential meaning missing in ethically bad phenomena)" (26). The hermeneutical element draws on Ricœur's theory of interpretation and aims to move from what the text says to what meaning we can understand by performing a closer reading (27).

Participants and setting

Six UK hospitals were included in the larger project for diversity in type of hospital (university/non university) and size of intensive care unit (ICU). All consultants from departments likely to refer patients to ICU were emailed an invitation to participate from the hospital's principal investigator. The goal was to recruit five consultants at each hospital. At hospitals with more than five volunteers, purposive sampling was used to achieve a spread of gender and clinical speciality. At two hospitals, where fewer than five volunteered, the principal investigators targeted consultants who were known to have opinions regarding intensive care referrals by sending a personal reminder. The inclusion process resulted in 27 consultants choosing to participate. For demographic data see Table 1.

For peer review only

Table 1. Demographic information for the participants

Characteristics (*n*=27)		
Gender, *n* (%)		
	Male	19 (70)
	Female	8 (30)
Age, mean (range)		47 (31–59)
Years of experience, mean (range)		
	In current specialty	10 (0.2–22)
	Since graduation	22 (8–34)
Specialty, *n* (%)		
	Medical specialties	15 (56)
	Surgical specialties	7 (26)
	Acute/Emergency Medicine	5 (18)

Ethical considerations

The project received ethics approval from the NHS Health Research Authority, and West Midlands-Coventry & Warwickshire research ethics committee (15/WM/0025, IRAS project ID 160660). Participants gave written consent prior to the interview. The overall project had a lay advisory group and two PPI co-investigators who contributed to the design, conduct and reporting of all aspects of the study. They did not contribute to the analysis of this data sub–set but were involved in analysis of other elements of the larger project.

Data collection

MS interviewed all but eight participants; the remaining were interviewed by AS or FG. Interviews were semi-structured and conducted face-to-face. Participants were initially asked whether they had referred or considered referring any patients to intensive care during the previous three weeks and were encouraged to describe their experiences of making the decision about whether to contact the intensive care team. Probes explored reasons for contacting ICU, the context of the process, and its outcome. They were also asked about their general experience of the referral process and how it could be improved. Interviews were audio-recorded and transcribed verbatim; average duration was 35 minutes (range 14–59 minutes).

Data analysis

The analysis included three steps; naïve reading, structural analysis, and comprehensive understanding, which were intertwined in a dialectic movement (26). All transcripts were read repeatedly and notes were made according to the whole. This initial interpretation of the whole of the interviews was formulated into a naïve understanding, but iteratively revised

For peer review only - http://bmjopen.bmj.com/site/about/guidelines.xhtml

during the analysis process. During the structural analysis, the data were initially coded into meaning units and grouped according to their meaning content. The meaning units were condensed and themes and sub-themes that shared similar meanings within the overall aim of the analysis were developed. Meaning units, sub-themes and themes were continuously validated and refined by returning to the data as well as the naïve understanding. As the analysis unfolded, a deeper understanding of the meaning of the participants' lived experiences was captured through shifting between the descriptive and interpretative levels of the analysis. The software program NVivo-11 facilitated the initial analysis process. During the writing up of the structural analysis, re-categorisation continued in light of the whole dataset. In the final step of the analysis, a comprehensive understanding with a further interpretation of the text was developed using the theory of the "Ethics of proximity" by Emmanuel Levinas (28), and the moral epistemology of Margaret urban Walker (29), to shed further understanding on the text (29-32). Throughout the process, analysis meetings were held between the co-authors to validate the findings. The SRQR reporting guideline was applied (33).

RESULTS

Naïve reading

The initial interpretation, iteratively revised, revealed that caring for a critically ill patient implies an obligation to urgently respond and take measures to secure a decision for the further care of the patient. The referring doctors experience the critically ill patient as being seriously affected by their illness, and the clinical situation is imprinted with the potential for death and suffering. They are aware of the consequences that their decisions will have on the patient's future life and the moral responsibility of their decision is evident.

Making judgments about referral to ICU is based on reflection about whether this escalation would be meaningful for the patient regarding outcome and future care trajectory. When there is certainty about the benefit of intensive care treatment, referrals appear to be straightforward for the clinicians. When they are convinced that palliative care or ward-based care is most appropriate for the patient, they do not ponder over referral to ICU at all. However, they struggle with making decisions for patients who are experienced as being in a 'grey area', where the meaningfulness of further care is difficult to interpret and imprinted with uncertainty and doubts. This implies not being convinced whether escalation to intensive care would be good and right for the patient while at the same time not being confident with making a decision to provide ward-based care alone.

When the decision-making process is loaded with doubts, the way in which the process unfolds is of moral significance. A profound understanding of the critically ill patient's clinical situation, as well as the troubled referring doctor, emerges in the encounter between the doctors and the patient and/or their family.

For peer review only - http://bmjopen.bmj.com/site/about/guidelines.xhtml

Structural analysis

The structural analysis revealed the themes ‘Burdensome doubt’, ‘Emerging understanding’ and ‘Responding to moral duties’ (Table 2).

Table 2. Themes and sub-themes.

Themes	Burdensome doubt	Emerging understanding	Responding to mutual moral duties
Subthemes	Being affected by the gravity of critical illness	Comprehension by seeing the patient at the bedside	Meeting in person to acknowledge concern
	Pondering about what is meaningful for the patient	Requiring senior experience to grasp clinical complexity	Protecting the patient through advocacy
	Ambivalence in searching for the patient’s voice	Needing engagement for broadened perspectives	Building relationships for confidence

Theme: Burdensome doubt

Burdensome doubt implies a struggle in making the right decision for the critically ill patient under difficult conditions while being highly aware of the risks of death and suffering. The referring doctors are affected by the gravity of critical illness. They ponder over whether escalation of care to an ICU would be good and meaningful for the patient and they experience ambivalence in searching for the important, but often ambivalent, voice of the patient. The burdensome doubt of the decision-making process signifies a need of trust in the process.

Being affected by the gravity of critical illness means that the referring doctors are burdened by a decision-making process that often is perceived as complex and difficult, imprinted with urgency and precariousness. The patients are perceived as being exhausted and often very affected by their critical condition and the process signifies a dimension of gravity and seriousness that places a moral responsibility on the referring doctor to make the right decision about the further care of the patient.

“It was an extremely difficult decision … For me, admitting her to the intensive care unit and supporting her respiratory system was as much about the symptomatology and control and actually good palliative care rather than … to turn this around. … that the patient’s very

For peer review only - http://bmjopen.bmj.com/site/about/guidelines.xhtml

conscious and gets very breathless, very worked up, very anxious and very frightened." (Haematology consultant, Hospital 2)

Pondering about what is meaningful for the patient refers to considering whether further escalation to intensive care would be good and beneficial for the patient. The pondering implies initially to obtain a clinical overview of the patient's medical situation in the face of an often rapid change in their condition. The clinical scenario is often experienced as being unclear with a lack of information and, by trying to articulate the precise clinical needs of the patient, they could ponder what intensive care could offer the patient.

"I can do lots of technological things but I'm not sure it gives people a good death and I'm not sure it gives people a good quality of life for their last few weeks." (Internal Medicine consultant, Hospital 2)

Ambivalence in searching for the patient's voice involves experiences of uncertainty in the involvement of the patient and family in the decision-making process. The doctors acknowledge on the one hand that the patient and family are natural partners in the process, but, on the other hand, their voice is experienced as ambiguous. The doctors reflect on the patient and family's ability to understand the different possibilities for the further care. The patients are often too sick and exhausted to voice anything at all and the doctors reflect on their ability to understand the different possibilities for their further care. They reflect on how patient and family´s wishes for treatment could be driven by fear as well as a lack of realism about understanding what intensive care could achieve.

"We should try and involve the patient, [but] she was clearly not in a position to be asked. We should try and ascertain her current and previous wishes. She was unable to give her current wishes … So we discussed with the family what they think she would have wanted and I think they were unsure as well." (Geriatric consultant, Hospital 2)

Theme: Emerging joint understanding

The decision-making process implies multiple perspectives and the process needs to stimulate an emerging understanding of the patient's precarious situation from the perspectives of the different stakeholders. Understanding emerges through comprehending the clinical situation at the bedside, through the eyes of experienced clinicians on a consultant level, as well as through engagement with broadened perspectives on the patient's situation. A profound understanding of the critical illness situation is essential for developing trust in decisions about the further care of the patient.

Comprehension by seeing the patient at the bedside means that the bedside encounter with the patient contextualizes the medical information that is available from multiple other sources, such as files, tests and examinations, and, thereby, the unique situation of the patient can emerge. Seeing the patient at the bedside means gaining a comprehensive understanding of the patient's situation. The importance of this extends to both the referring doctor and the intensivist.

For peer review only - http://bmjopen.bmj.com/site/about/guidelines.xhtml

"First of all, undoubtedly the reality that it's a person, you see them, so if somebody phones me it's a list of numbers, blood pressure, observations I get because I'm a kidney physician. Whereas when you go to see somebody, they're real, they've got family and when you're then thinking about it you contextualize it, you think about this is a real person." (Nephrology consultant, Hospital 3)

Patients could be in more substantial need of intensive care than the numerical physiological measures and telephone reports have suggested. Initial reluctance from the intensivist to admit a patient can rapidly change when they see the patient. When intensivists dismiss the need for admission to intensive care without seeing the patients, the referring doctors regard this as irresponsible practice.

Requiring experience on consultant level for both the referring doctor and the intensivist refers to being able to grasp clinical complexity and to understand critical illness as a dynamic condition. Being able to anticipate the unpredictable in the situation implies being able to think upfront about the further care of the patient to secure the safety of the patient as well as the referring doctor's own confidence in the unpredictable situation.

"Especially when it's the patients that could get a lot worse, it's almost a seniority thing. They [junior doctors] don't know enough, haven't seen enough to realize that the information that you're imparting is because we're basing it on experience of things going worse." (Acute and Emergency Medicine consultant, Hospital 1)

Needing engagement for broadened perspectives means a mutual responsibility of the referring doctor and the intensivist to actively seek more information about the patient's situation to broaden their own perspectives and contribute to a more profound understanding of the patient's situation.

Referring doctors describe their need to obtain the perspective from intensive care on what can be done as well as a realistic view on what it is possible to do for a critically ill patient. They describe how they experience only being able to judge the most acute needs and acknowledge the clinical situation over a short period of time and that the intensivist's judgment contributes a broader perspective by providing new insights and understanding. The intensivist is experienced as an important sounding board for the referring doctor.

"You're not quite sure, you just know someone's ill and you're just trying to work out what is it that I need and then that consultant who I spoke to is very good at sort of saying: 'Well what is it you're actually asking for? Let's work this out!'" (Internal Medicine consultant, Hospital 1)

However, experiences of lack of engagement from the intensivist and a too-narrow perspective on the patient's situation means a lack of understanding of the patient's difficult situation and an increased burden on the referral doctor with loss of trust.

"That's a very easy thing to do, to walk in and say: 'Not fit for intensive care,' then wander back to your intensive care unit. That's quite an easy decision to make. You're not then the

For peer review only - http://bmjopen.bmj.com/site/about/guidelines.xhtml

person who's there, dealing with that patient, that family saying: 'Why aren't you treating my mum?'" (Colorectal surgeon, Hospital 5)

Theme: Responding to mutual moral duties

Fulfilling moral duties is part of the process of gaining trust in the decision and this is achieved in the encounter between the doctors. The referring doctor and the intensivist meeting in person during the process gives possibilities for acknowledging concern to the referring doctor in the difficult situation of negotiating care for a critically ill patient. The referring doctors experience a duty to protect their patients from harm and to advocate for their needs of further intensive care. They also acknowledge their own role in the relationship with their intensivist colleagues and how they have a responsibility to contribute to instilling confidence in the process.

Meeting in person to acknowledge concern refers to the need to meet face-to-face with the intensivist in the decision-making process. Meeting in person facilitates the discussion when dealing with the many aspects of complex decisions, new questions are answered directly, and it is easier to make a further plan for the patient's care together. The referring doctor's concern for the critically ill patient can be acknowledged and more confidence in the decision can be gained by sharing the burden of making complex decision.

"People who come to see the patient, make a proper assessment, talk to me and explain to me, I can ask questions and we come to an agreement. I think that's straightforward. There the trust grows very quickly." (Respiratory physician, Hospital 6)

Protecting the patient through advocacy means that the referring doctors see themselves as having a duty to advocate on behalf of the patient when there is disagreement and lack of confidence in the process about the further care of the patient. This includes arguing for the possible benefit of intensive care if they are convinced that this would be the right decision.

Advocacy is seen as being important when bed capacity is a concern. The referring doctors are aware of the capacity issues regarding intensive care. However, they see that their obligation in the decision-making process is to strictly focus on the needs of their critically ill patient while not being influenced by the availability of beds.

"Beds are tough but, that's not a part of the decision-making; is the patient going to get better care on ITU than where they are now? Can we do something to intervene and then if the decision-making is 'yes' and if appropriate for the patient then the final [a bed] is secondary." (Haematology consultant, Hospital 6)

Being the patient's advocate also involves arguing for the time needed in ICU to get a clearer picture of the medical condition. Admission with a restriction in length of stay or type of intervention could be offered to give the patient a chance, but at the same time restricts long-term suffering. These negotiations are a way of operationalizing the duty of protection from harm.

For peer review only - http://bmjopen.bmj.com/site/about/guidelines.xhtml

Building relationships for confidence refers to the significance of the relationship between the referral doctor and the intensivist in gaining confidence in the process and further trust in the decision. The referring doctors describe their working relationship with the intensivist as being important for their care of the patients and for the decision-making process. Building the relationship is achieved through learning to know each other over time while sharing care for critically ill patients. Although the intensivist makes the formal decision about an intensive care admission, the referral doctors describe their own duties in making discerning and adequate clinical judgements to contribute to a necessary and mutual confidence in the process.

A confident relationship between them could mean that the referring doctors ignore the formal referral system and directly contact senior intensive care colleagues to discuss their patients. The referral doctors also describe having a personal and extended responsibility for patients being cared for on the intensive care unit. This responsibility could be for patients who they have known for a long time, but also patients suffering from complicated or rare diseases, where they regard their knowledge and experience to be important. This responsibility is grounded in a personal relationship of mutual confidence and support that the referral doctors are eager to foster.

"I have a good relationship with the more senior ITU consultants and I suppose it all is dependent on trust. I trust their judgement and they trust my judgement and that is based on years of working with each other and seeing what we do and how we make decisions … it comes from experience, so if I referred patients to the intensive care unit and they took them and then felt time and time again they weren't appropriate referrals they would stop trusting my judgement." (Nephrology consultant, Hospital 3)

Comprehensive understanding: Needing trust nurtured through responsible proximity

The structural analysis revealed the themes of the burdensome doubt of the referring doctor, how to contribute to an emerging, joint understanding of the situation and mutual moral duties that need response. When the clinical scenario does not have an obvious right or wrong answer to what ought to be done, *the way* in which the decision is made and *how* the process unfolds, is of moral significance for gaining trust in the process. Trust is necessary and needed in the decision-making process and can be nurtured in a relationship characterized by a responsible proximity between the stakeholders; the referring doctor, the patient and the intensivist. The further comprehensive understanding of the data was developed and further interpreted in light of the writings of Emmanuel Levinas and the concept of 'Ethics of proximity' and the moral epistemology of Margaret Urban Walker (28, 29).

The face-to-face encounter, and the moral responsibility that manifests itself in this encounter, is central to Levinas' philosophy (30). It deals with the existential character of human relations and signifies a search for deeper patterns of being human in relation to others. Levinas describes how, in the encounter with 'The Other's Face', physical presence is not foremost; rather, it is the precognitive of being called by that 'other' and responding to that 'otherness'. The Other is substantially different from me and this otherness establishes a basic

For peer review only - http://bmjopen.bmj.com/site/about/guidelines.xhtml

asymmetry in this particular relation between us. Based on the asymmetrical relation of otherness as well as proximity, comes, according to Levinas, the moral claim of protection and responsibility.

Seeing the critically ill patient in person is a necessity in the decision-making process. This claim comprises both the referring doctor as well as the intensivist. Seeing the patient is important for establishing an understanding of the patient's situation and the emerging understanding of the clinical options. Seeing the patient could be further interpreted, in light of Levinas' philosophy, as being an expression of the demand to encounter the critically ill patient as The Other's Face, and so recognize, through a clinical proximity, the moral claim for responsibility in a critical and vulnerable situation. Seeing the face of the patient could be understood as adding substantial moral information to the process – what is at stake and what ought to be done – over and above the medical information.

In the asymmetrical relation with The Other, we encounter, according to Levinas, The Other's, as well as our own, vulnerability, and the empirical findings reveal what could be interpreted as a substantial vulnerability (31). The critically ill patient is perceived as being vulnerable, imprinting gravity in the decision-making process. The referring doctor's burdensome doubt exposes their own vulnerability – being close to the existential dimensions of life and burdened by the seriousness and uncertainty of the decision. The decision-making process itself could also be seen as being vulnerable in being constrained by a lack of time and information, and with substantial uncertainty and doubts, pervaded with possibilities of disregarded moral duties and mistrust.

Levinas describes how, in the encounter with The Other's Face, we are called to protect and take responsibility. This is in line with the referring doctors' advocacy for the patient when they think the patient should be admitted to the ICU and could be seen as a clinical outlook of protection and responsibility. However, the moral claim of responsibility and protection will not in itself inform which decision to make or what is the right thing to do, but could be seen as an underlying prerequisite for making a moral judgement, through clinical and moral proximity, for assuming a mutual, moral responsibility in the situation and finally nurturing trust.

Proximity in ethics can be understood, according to Levinas, as a phenomenological description of an underlying structure of humans and not foremost as a normative theory for ethics, and relates to the possibilities as well as problems with the normativity of proximity in ethics (32). Proximity in ethics has been described as an important part of moral perception. Moral perception is used to describe our capacity to acknowledge morally salient aspects of a particular situation (34). Moral perception, to see that something matters morally, can be distinguished from moral reasoning, a more conscious deliberation about action and reasons for actions (35). In the present study, the importance of clinical proximity could therefore be interpreted as being important for the moral perception of a critical situation. Being close to the patient, the moral obligation of the doctors to always see the patient at the bedside, could contribute to identifying morally salient aspects of the patient's situation that are not detectable over the phone or in notes and could be seen as a component of nurturing trust.

For peer review only - http://bmjopen.bmj.com/site/about/guidelines.xhtml

Proximity in ethics has, however, been questioned for being a threat to a necessary distance to be able to act ethically (32). Traditionally, ethical theories have emphasized the moral agent as an independent, impartial and rational person who approaches moral problems by prioritizing and balancing principles through an empirical or scientific inquiry (36, 37). However, since the 1980s, ethics of care has offered a different perspective where it is the interpersonal and relational connectedness between humans that has moral relevance (38). Rather than arguing for impartial moral judgments, ethics of care takes into account the web of relationships that feature all humans, patients as well as health care professionals, and, accordingly, dependency and vulnerability (39). The implications for moral judgements are that moral reasoning is more about trying to harmonize competing interests and perspectives rather than applying moral principles impartially on a difficult situation.

The importance of relationships for morality is the starting point for the philosopher Margaret Urban Walker's moral epistemology. She considers the web of relationships, in which we are all embedded, as being fundamental for our understanding of morality (29). She argues that morality is best understood as "a socially embodied medium of mutual understandings and negotiation between people over their responsibility for things open to human care and responses" (29). Morality consists of practices, not theories, and could be seen as practices of responsibility in which content could be found by tracking the responsibilities between human beings. The meanings of the lived experiences of the referral doctors' experiences of the decision-making process could, in light of this, be further understood as a moral encounter where the patient's need of care and a human response is the point of departure for the negotiation between the referring doctor and intensivist to develop a mutual understanding of the patient's situation and negotiate further responsible and meaningful care.

Urban Walker (29) describes how the content of morality could be found by tracking the moral responsibilities in the web of relationships. Looking at the structural analysis of our data from this perspective, experiences of sole moral duties are described, but most of them are described as shared and mutual, embracing both the referring doctor and the intensivist. The referring doctors describe their sole moral responsibilities to involve patient and family in the decision-making process and their obligation to advocate for their patient's needs of further intensive care if they are convinced that it would be the right decision. Most of the referring doctors' lived experiences of the decision-making process could, however, be understood as shared moral responsibilities which are situated in the web of relationships between them and the intensivists. They both need to see the patient at the bedside, to increase their understanding of the precarious situation. The decision-making process should be accomplished between two consultants because seniority is crucial to being able to understand complexity. Both of the doctors need to engage with each other and to strive for broadened perspectives. They should meet face-to-face in the decision-making process and work for mutual confidence in their relationship. These descriptions could be seen as moral traits necessary for nurturing trust in the often difficult decision-making process.

For peer review only - http://bmjopen.bmj.com/site/about/guidelines.xhtml

METHODOLOGICAL CONSIDERATIONS

The data were not collected with the intention of using a phenomenological hermeneutic analysis method, which could pose a potential threat to validity. However, we regarded the data as being rich with the possibility of capturing meanings of lived experiences. The first author, who led the analysis, did not conduct any interviews, which could be seen as a weakness. However, the data analysis process was conducted under constant critical review and reflection by the co-authors, who were involved in the project (MS, AS, FG) and from outside the project (AB), ensuring reliability and trustworthiness.

It can be argued that the sample size was relatively large for this method of analysis, thus challenging the depth of the analysis when dealing with the large quantity of data, but it may also be regarded as a strength, as we captured varied narratives from different clinical specialties as well as types of hospital.

The data were gathered in a UK context. The clinical context of the study, caring for patients where further intensive care is imprinted with doubts and uncertainties, is present in many developed countries. In spite of cultural and organizational differences, the importance of trust in relations of proximity would most likely be of relevance and thus transferable to other countries and cultural contexts.

CONCLUSION

The interviews with the consultant doctors about their lived experiences of the decision-making process when referring patients to intensive care revealed the importance of trust. A trusted decision-making process is engendered by a responsible proximity between the stake-holding doctors to assume and support their moral responsibility for the further care of the critically ill patient.

The study has clinical implications in how guidance to support the process of referral to intensive care should be framed to support clinicians. Education and training at all levels must attend to the negotiation of the burdensome doubts and uncertainties in the referral process. The healthcare system must facilitate this through organizational and leadership structures in the fostering of relationships between the patients and their healthcare providers, in order to nurture trust and protect the vulnerability of human beings.

During the last months, the COVID-19 pandemic has showed us the extensive vulnerability of critical ill patients as well as the substantial need for trust, not only between clinical stakeholders, but also between policymakers and the general public alike.

For peer review only - http://bmjopen.bmj.com/site/about/guidelines.xhtml

Author statement:

Kaja Heidenreich; conducted the coding, led the analysis and wrote the manuscript.

Anne Slowther; main project leader, conducted two interviews, scrutinized the analysis and reviewed the manuscript.

Frances Griffiths, leader of the project work-package in which data were collected, conducted six interviews and reviewed the manuscript.

Anders Bremer; provided methodological support for the phenomenological-hermeneutical analysis, scrutinized this analysis, and reviewed the manuscript.

Mia Svantesson; conducted nineteen interviews, scrutinized the analysis and reviewed the manuscript.

We are grateful to the consultants who participated in the study.

REFERENCES

1. Brinkman S, de Jonge E, Abu-Hanna A, Arbous MS, de Lange DW, de Keizer NF. Mortality after hospital discharge in ICU patients. Crit Care Med. 2013;41(5):1229-36.
2. Williams TA, Dobb GJ, Finn JC, Knuiman MW, Geelhoed E, Lee KY, et al. Determinants of long-term survival after intensive care. Crit Care Med. 2008;36(5):1523-30.
3. Montuclard L, Garrouste-Orgeas M, Timsit JF, Misset B, De Jonghe B, Carlet J. Outcome, functional autonomy, and quality of life of elderly patients with a long-term intensive care unit stay. Crit Care Med. 2000;28(10):3389-95.
4. ICNARC. Key statistics from the Case Mix Programme - Adult, general critical care units 1, April 2015 to 31 March 2016. 2017.
5. Mnatzaganian G, Galai N, Sprung CL, Zitser-Gurevich Y, Mandel M, Ben-Hur D, et al. Increased risk of bloodstream and urinary infections in intensive care unit (ICU) patients compared with patients fitting ICU admission criteria treated in regular wards. J Hosp Infect. 2005;59(4):331-42.
6. Azoulay E, Pochard F, Kentish-Barnes N, Chevret S, Aboab J, Adrie C, et al. Risk of post-traumatic stress symptoms in family members of intensive care unit patients. Am J Respir Crit Care Med. 2005;171(9):987-94.
7. Garrouste-Orgeas M, Perier A, Mouricou P, Gregoire C, Bruel C, Brochon S, et al. Writing in and reading ICU diaries: qualitative study of families' experience in the ICU. PLoS One. 2014;9(10):e110146.
8. Wade DM, Howell DC, Weinman JA, Hardy RJ, Mythen MG, Brewin CR, et al. Investigating risk factors for psychological morbidity three months after intensive care: a prospective cohort study. Crit Care. 2012;16(5):R192.
9. Storli SL, Lindseth A, Asplund K. A journey in quest of meaning: a hermeneutic-phenomenological study on living with memories from intensive care. Nurs Crit Care. 2008;13(2):86-96.
10. Svenningsen H, Egerod I, Dreyer P. Strange and scary memories of the intensive care unit: a qualitative, longitudinal study inspired by Ricoeur's interpretation theory. J Clin Nurs. 2016;25(19-20):2807-15.
11. Bassford C, Griffiths F, Svantesson M, Ryan M, Krucien N, Dale J, et al. Gatekeeping in intensive care: Understanding and improving the decision-making process surrounding admission to the intensive care unit. University Hospitals Coventry & Warwickshire NHS Trust; 2018.

For peer review only - http://bmjopen.bmj.com/site/about/guidelines.xhtml

12. Uy J, White DB, Mohan D, Arnold RM, Barnato AE. Physicians' decision-making roles for an acutely unstable critically and terminally ill patient. Crit Care Med. 2013;41(6):1511-7.
13. McNarry AF, Goldhill DR. Intensive care admission decisions for a patient with limited survival prospects: a questionnaire and database analysis. Intensive Care Med. 2004;30(2):325-30.
14. Einav S, Soudry E, Levin PD, Grunfeld GB, Sprung CL. Intensive care physicians' attitudes concerning distribution of intensive care resources. A comparison of Israeli, North American and European cohorts. Intensive Care Med. 2004;30(6):1140-3.
15. Garrouste-Orgeas M, Tabah A, Vesin A, Philippart F, Kpodji A, Bruel C, et al. The ETHICA study (part II): simulation study of determinants and variability of ICU physician decisions in patients aged 80 or over. Intensive Care Med. 2013;39(9):1574-83.
16. Nates JL, Nunnally M, Kleinpell R, Blosser S, Goldner J, Birriel B, et al. ICU Admission, Discharge, and Triage Guidelines: A Framework to Enhance Clinical Operations, Development of Institutional Policies, and Further Research. Crit Care Med. 2016;44(8):1553-602.
17. Cooper AB, Joglekar AS, Gibson J, Swota AH, Martin DK. Communication of bed allocation decisions in a critical care unit and accountability for reasonableness. BMC Health Serv Res. 2005;5:67.
18. Mielke J, Martin DK, Singer PA. Priority setting in a hospital critical care unit: qualitative case study. Crit Care Med. 2003;31(12):2764-8.
19. Giannini A, Consonni D. Physicians' perceptions and attitudes regarding inappropriate admissions and resource allocation in the intensive care setting. Br J Anaesth. 2006;96(1):57-62.
20. Blanch L, Abillama FF, Amin P, Christian M, Joynt GM, Myburgh J, et al. Triage decisions for ICU admission: Report from the Task Force of the World Federation of Societies of Intensive and Critical Care Medicine. J Crit Care. 2016;36:301-5.
21. Young PJ, Arnold R. Intensive care triage in Australia and New Zealand. N Z Med J. 2010;123(1316):33-46.
22. Pintado MC, Villa P, Gonzalez-Garcia N, Lujan J, Molina R, Trascasa M, et al. Characteristics and outcomes of elderly patients refused to ICU. TheScientificWorldJournal. 2013;2013:590837.
23. Iapichino G, Corbella D, Minelli C, Mills GH, Artigas A, Edbooke DL, et al. Reasons for refusal of admission to intensive care and impact on mortality. Intensive Care Med. 2010;36(10):1772-9.
24. Oerlemans AJ, van Sluisveld N, van Leeuwen ES, Wollersheim H, Dekkers WJ, Zegers M. Ethical problems in intensive care unit admission and discharge decisions: a qualitative study among physicians and nurses in the Netherlands. BMC Med Ethics. 2015;16:9.
25. Halvorsen K, Forde R, Nortvedt P. Value choices and considerations when limiting intensive care treatment: a qualitative study. Acta Anaesthesiol Scand. 2009;53(1):10-7.
26. Lindseth A, Norberg A. A phenomenological hermeneutical method for researching lived experience. Scand J Caring Sci. 2004;18(2):145-53.
27. Ricoeur P. Interpretation theory, discourse and the surplus of meaning. Fort Worth, Texas: TCU Press; 1976.
28. Levinas E. Totality and Infinity. An Essay on Exteriority. Pittsburgh: Duquesne University Press; 1969.
29. Urban Walker M. Moral understandings, a feminist study in ethics. New York: Oxford University Press; 2007.
30. Levinas E. Ethics and Infinity. Conversations With Philippe Nemo. Pittsburgh: Duquesne University Press; 1985.
31. Bergo B. Emmanuel Levinas. In: Zalta E, editor. Stanford Encyclopedia of Philosophy. Fall 2017 Edition ed. Stanford: The Metaphysics Research Lab, Center for the Study of Language and Information, Stanford University; 2017.
32. Nortvedt P, Nordhaug M. The principle and problem of proximity in ethics. J Med Ethics. 2008;34(3):156-61.

For peer review only - http://bmjopen.bmj.com/site/about/guidelines.xhtml

33. O'Brien BC, Harris IB, Beckman TJ, Reed DA, Cook DA. Standards for reporting qualitative research: a synthesis of recommendations. Acad Med. 2014;89(9):1245-51.
34. Blum L. Moral perception and particularity. New York: Cambridge University Press; 1994.
35. Richardson HS. Moral Reasoning. In: Zalta EN, editor. The Stanford Encyclopedia of Philosophy. Winter 2014 ed: The Metaphysics Research Lab, Center for the Study of Language and Information, Stanford University; 2014.
36. Beauchamp TL, Childress JF. Principles of biomedical ethics. 7th ed. New York: Oxford University Press; 2013.
37. Gillon R. An introduction to philosophical medical ethics. Chichester: England: John Wiley & sons; 1985.
38. Nodding N. Caring: A Feminine Approach to Ethics and Moral Education. Berkeley: University of California Press; 1984.
39. Nortvedt P, Hem MH, Skirbekk H. The ethics of care: role obligations and moderate partiality in health care. Nurs Ethics. 2011;18(2):192-200.

For peer review only

For peer review only - http://bmjopen.bmj.com/site/about/guidelines.xhtml

Reporting checklist for qualitative study based on the SRQR guidelines

Title and abstract

Item 1 See Title page

Item 2 See Abstract

Problem formulation

Item 3 See “Background” page 3.

Purpose or research question

Item 4 See “Background” page 3.

Qualitative approach and research paradigm

Item 5 See “Design” page 4

Researcher characteristics and reflexivity

Item 6 See “Author statement” page 14

Context

Item 7 See “Participants and setting” page 4

Sampling strategy

Item 8 See “Participants and setting” page 4

Ethical issues pertaining to human subjects

Item 9 See “Ethical considerations” page 5

Data collection method

Item 10 See “Data collection” page 5

Data collection instruments and technologies

Item 11 See “Data collection” page 5

Units of study

Item 12 See “Participants and setting” page 4 and Table 1

Data processing

Item 13 See “Data analysis” page 5-6

For peer review only

Data analysis

Item 14 See “Data analysis” page 5-6

Techniques to enhance trustworthiness

Item 15 See “Data analysis” page 5-6

Syntheses and interpretation

Item 16 See “Comprehensive understanding” page 11-13

Links to empirical data

Item 17 Quotes are embedded in the result section to illustrate the findings.

Integration with prior work, implications, transferability and contribution(s) to the field

Item 18 See “Comprehensive understanding” page 11-13

Limitations

Item 19 See “Strengths and limitations” on page 1

Conflict of interest

Item 20 See page 2

Funding

Item 21 See page 1

For peer review only

BMJ Open

UK Consultants experiences of the decision-making process around referral to intensive care: an interview study

Journal:	*BMJ Open*
Manuscript ID	bmjopen-2020-044752.R1
Article Type:	Original research
Date Submitted by the Author:	25-Feb-2021
Complete List of Authors:	Heidenreich, Kaja; University Health Care Research Center, Faculty of Medicine and Health;<br>Slowther, Anne-Marie; University of Warwick, Warwick Medical School<br>Griffiths, Frances; University of Warwick, Warwick Medical School<br>Bremer, Anders; Linnaeus University, Faculty of Health and Life Sciences, Department of Health and Caring Sciences<br>Svantesson, Mia; Orebro University, Centre for Healtcare Sciences Faculty of Health and Medicine
**Primary Subject Heading**:	Intensive care
Secondary Subject Heading:	Ethics, Qualitative research
Keywords:	MEDICAL ETHICS, QUALITATIVE RESEARCH, Adult intensive & critical care < INTENSIVE & CRITICAL CARE

SCHOLARONE™
Manuscripts

I, the Submitting Author has the right to grant and does grant on behalf of all authors of the Work (as defined in the below author licence), an exclusive licence and/or a non-exclusive licence for contributions from authors who are: i) UK Crown employees; ii) where BMJ has agreed a CC-BY licence shall apply, and/or iii) in accordance with the terms applicable for US Federal Government officers or employees acting as part of their official duties; on a worldwide, perpetual, irrevocable, royalty-free basis to BMJ Publishing Group Ltd ("BMJ") its licensees and where the relevant Journal is co-owned by BMJ to the co-owners of the Journal, to publish the Work in this journal and any other BMJ products and to exploit all rights, as set out in our licence.

The Submitting Author accepts and understands that any supply made under these terms is made by BMJ to the Submitting Author unless you are acting as an employee on behalf of your employer or a postgraduate student of an affiliated institution which is paying any applicable article publishing charge ("APC") for Open Access articles. Where the Submitting Author wishes to make the Work available on an Open Access basis (and intends to pay the relevant APC), the terms of reuse of such Open Access shall be governed by a Creative Commons licence – details of these licences and which Creative Commons licence will apply to this Work are set out in our licence referred to above.

Other than as permitted in any relevant BMJ Author's Self Archiving Policies, I confirm this Work has not been accepted for publication elsewhere, is not being considered for publication elsewhere and does not duplicate material already published. I confirm all authors consent to publication of this Work and authorise the granting of this licence.

For peer review only

UK Consultants experiences of the decision-making process around referral to intensive care: an interview study

Kaja Heidenreich [1]

Anne Slowther [2]

Frances Griffiths [2]

Anders Bremer [3]

Mia Svantesson [1]

1) University Health Care Research Centre, Faculty of Medicine and Health, Örebro University, Örebro, Sweden
2) Division of Health Sciences, Warwick Medical School, University of Warwick, UK
3) Faculty of Health and Life Sciences, Linnaeus University, Växjö, Sweden

Corresponding author:

Kaja Heidenreich

University Health Care Research Centre
S-huset vån 2
SE-701 85 Örebro
Sweden

Telephone +46 70 269 4705

E-mail: kaja.heidenreich@regionorebrolan.se

Kaja Heidenreich: PhD Student, University Health Care Research Center, Faculty of Medicine and Health, Örebro University, Sweden
Anne Slowther: Professor of Clinical Ethics, Warwick Medical School, University of Warwick, UK
Frances Griffiths: Professor of Medicine in Society, Warwick Medical School, University of Warwick, UK
Anders Bremer: Senior Lecturer, Associate professor, Faculty of Health and Life Sciences, Linnaeus University, Växjö, Sweden
Mia Svantesson: Senior Research Fellow, Associate professor, University Health Care Research Center, Faculty of Medicine and Health, Örebro University, Sweden

Word count: 5175 words (4683)

Key words: Critical care, Clinical Decision-making, Clinical Ethics, Ethical Theory, Physicians.

For peer review only - http://bmjopen.bmj.com/site/about/guidelines.xhtml

ABSTRACT

Objectives: The decision whether to initiate intensive care for the critically ill patient involves ethical questions regarding what is good and right for the patient. It is not clear how referring doctors negotiate these issues in practice. The aim of this study was to describe and understand consultants' experiences of the decision-making process around referral to intensive care.

Design: Qualitative interviews were analysed according to a phenomenological hermeneutical method.

Setting and participants: Consultant doctors (n=27) from departments regularly referring patients to intensive care in six UK hospitals.

Results: In the precarious and uncertain situation of critical illness, trust in the decision-making process is needed and can be enhanced through the way in which the process unfolds. When there are no obvious right or wrong answers as to what ought to be done, how the decision is made and how the process unfolds is morally important. Through acknowledging the burdensome doubts in the process, contributing to an emerging, joint understanding of the patient's situation, and responding to mutual moral duties of the doctors involved, trust in the decision-making process can be enhanced and a shared moral responsibility between the stake holding doctors can be assumed.

Conclusions: The findings highlight the importance of trust in the decision-making process and how the relationships between the stake-holding doctors are crucial to support their moral responsibility for the patient. Poor interpersonal relationships can damage trust and negatively impact decisions made on behalf of a critically ill patient. For this reason, active attempts must be made to foster good relationships between doctors. This is not only important to create a positive working environment, but a mechanism to improve patient outcomes.

For peer review only

Strengths and limitations of this study

- The phenomenological hermeneutic method enabled the meanings of the lived experiences of a morally complex decision-making process to be captured, resulting in a deeper understanding of this process.
- The sample included hospitals of different sizes and a range of clinical specialties, which provided a variety of experiences to inform the research question.
- The sample size was large for a phenomenological hermeneutical analysis, which challenged the analysis process and the depth of analysis.

For peer review only - http://bmjopen.bmj.com/site/about/guidelines.xhtml

BACKGROUND

Admission to an intensive care unit (ICU) allows critically ill patients to access life-saving treatment. However, the short- and long-term mortality of ICU patients is substantial (1-3). In the UK, 20% of patients admitted to the ICU die in the unit and, additionally, 10% will die before hospital discharge (4). The care received involves invasive, distressing and potentially harmful interventions, as well as psychological stress for both patients and their families (5-8). Patients' lived experiences, reported after a stay in the ICU, have revealed strange and frightening memories as well as a process of struggling to find meaning in their ongoing lives (9, 10).

Given the burden of intensive care therapy and the limited prognosis, intensive care treatment will not be beneficial for all patients, and ward-based care or palliative care might be more beneficial (11). The decision whether to refer and admit a patient to ICU is often complex, time pressured, and made in the context of clinical uncertainty with limited knowledge of the patient's wishes for their further care (11, 12). This complex situation was the departure for a multidimensional mixed methods project, "Understanding and improving the decision-making process surrounding admission to the intensive care unit", conducted at six NHS trusts in the Midlands, UK (11-14). The present paper is a sub-study of the wider project.

Previous research suggests that there is large variation in how decisions about admission to ICU are made, depending on the individual clinician, unit, hospital and country characteristics (15-18). The focus of previous research has been on justice and resource allocation (19-23) and factors that influence the decisions (24, 25). In a systematic review, factors that correlated a decision were bed availability, severity of illness, referral ward or team, patient choice, do not resuscitate status, age and functional baseline (26). Experiences of the decision-making *process* are less explored, but according to a recent systematic review of this literature, professional relationships, attitudes and communication between stakeholders as well as acknowledging uncertainty seemed important (27). However, the described experiences have foremost been considered from the perspective of intensive care and the perspective of referring clinicians is missing (28, 29). The Society of Critical Care Medicine offers guidelines for admission, discharge and triage, but only sparse guidance about the complex decision-making processes (30).

The wider project used a focussed ethnography method to describe and understand the decision-making process (11, 12). The project included the perspective of referring doctors from wards and emergency departments, as little is known about their experiences of the decision-making process. In addition, clinical experience is valuable in developing deeper knowledge about this complex decision-making process and, therefore, the perspective of consultant doctors was sought to develop further understanding of the process.

Thus, the aim of the present study was to describe and understand consultant doctors' experiences of the decision-making process around referral to intensive care.

For peer review only - http://bmjopen.bmj.com/site/about/guidelines.xhtml

METHOD

Design

Qualitative design, using phenomenological hermeneutics as analysis method (31). The present paper is an analysis of a sub-set of the data collected in the ethnographic study in the NIHR-project (11).

The phenomenological hermeneutical method was developed for the interpretation of lived experiences of ethically difficult situations, so-called "lived ethics" (26). "Lived ethics", i.e., how people act and think about real moral situations, are not easily accessible in empirical research because most people have profound difficulties explaining why their actions are morally good or bad. In this study, we have used the method to reveal the underlying ethics through interpreting the text through a dialectic movement between understanding and explanation (31). The phenomenological element aims to describe and understand the lived experiences of "actions, attitudes, relations or other human matters as ethically good or bad" (31). The hermeneutical element draws on Ricœur's theory of interpretation and aims to move from what the text says to what meaning we can understand by performing a closer reading (32).

Participants and setting

Six UK hospitals were included in the larger project for diversity in type of hospital (university/non university) and size of intensive care unit (ICU) (12). All consultants from departments likely to refer patients to ICU were emailed an invitation to participate from the hospital's principal investigator. The goal was to recruit five consultants at each hospital. At hospitals with more than five volunteers, purposive sampling was used to achieve a spread of gender and clinical speciality. At two hospitals, where fewer than five volunteered, the principal investigators targeted consultants who were known to have opinions regarding intensive care referrals by sending a personal reminder. The inclusion process resulted in 27 consultants choosing to participate. The participants represented medicine specialities (acute/emergency, general internal, respiratory, cardiology, renal, gastroenterology, haematology, endocrinology, infectious disease, neurology, geriatrics, oncology) and surgical specialties (general, colorectal, neurosurgery, orthopedics). For demographic data see Table 1.

Table 1. Demographic information for the participants

Characteristics (n=27)	
Gender, n (%)	
Male	19 (70)
Female	8 (30)
Age, mean (range)	47 (31–59)
Years of experience, mean (range)	
In current specialty	10 (0.2–22)

For peer review only - http://bmjopen.bmj.com/site/about/guidelines.xhtml

Since graduation	22 (8–34)
Specialty, *n* (%)	
Medical specialties	15 (56)
Surgical specialties	7 (26)
Acute/Emergency Medicine	5 (18)

Ethical considerations

The project received ethics approval from the NHS Health Research Authority, and West Midlands-Coventry & Warwickshire research ethics committee (15/WM/0025, IRAS project ID 160660). Participants gave written consent prior to the interview.

Patient and Public involvement

The overall project had a lay advisory group and two PPI co-investigators who contributed to the design, conduct and reporting of all aspects of the study. They did not contribute to the analysis of this data sub–set but were involved in analysis of other elements of the larger project.

Data collection

MS interviewed all but eight participants; the remaining were interviewed by AS or FG. Interviews were semi-structured and conducted face-to-face. Participants were initially asked whether they had referred or considered referring any patients to intensive care during the previous three weeks and were encouraged to describe their experiences of making the decision about whether to contact the intensive care team. Probes explored reasons for contacting ICU, the context of the process, and its outcome. They were also asked about their general experience of the referral process and how it could be improved. Interviews were audio-recorded and transcribed verbatim; average duration was 35 minutes (range 14–59 minutes). The interview guide could be found under supplemental files.

Data analysis

The analysis included three steps; naïve reading, structural analysis, and comprehensive understanding, which were intertwined in a dialectic movement (31). All transcripts were read repeatedly and notes were made according to the whole. This initial interpretation of the whole was formulated into a naïve understanding, but iteratively revised during the analysis process. During the second step, the structural analysis, the data were decontextualized in order to explain the text through coding into meaning units which were grouped according to their meaning content. The meaning units were condensed and themes and sub-themes that shared similar meanings within the overall aim of the analysis were developed. Meaning units, sub-themes and themes were continuously validated and refined by returning to the data as well as the naïve understanding. As the analysis unfolded, a deeper understanding of the meaning of the participants' lived experiences was captured through shifting between the

For peer review only - http://bmjopen.bmj.com/site/about/guidelines.xhtml

descriptive and interpretative levels of the analysis. Due to the extensive amount of data, the software program NVivo-11 was used to facilitate the initial analysis process. During the writing up of the structural analysis, re-categorisation continued in light of the whole dataset. In the final step of the analysis, a comprehensive understanding with a further interpretation of the text was developed using the theory of the "Ethics of proximity" by Emmanuel Levinas (33, 34).

Throughout the process, analysis meetings were held between the co-authors to validate the findings. The SRQR reporting guideline was applied (35).

RESULTS

Naïve reading

The initial interpretation of the text, iteratively revised, revealed that caring for a critically ill patient implies an obligation to urgently respond and take measures to secure a decision for the further care of the patient. The referring doctors experience the critically ill patient as being seriously affected by their illness, and the clinical situation is imprinted with the potential for death and suffering. They are aware of the consequences that their decisions will have on the patient's future life and the moral responsibility of their decision is evident.

Making judgments about referral to ICU is based on reflection about whether this escalation would be beneficial for the patient regarding outcome and future care trajectory. When there is certainty about the benefit of intensive care treatment, referrals appear to be straightforward for the clinicians and the referring process could have larger diversity. When they are convinced that palliative care or ward-based care is most appropriate for the patient, they do not ponder over referral to ICU at all.

However, they struggle with making decisions for patients who are experienced as being in a 'grey area', where the meaningfulness of further care is difficult to interpret and imprinted with uncertainty and doubts. This implies not being convinced whether escalation to intensive care would be good and right for the patient while at the same time not being confident with making a decision to provide ward-based care alone.

When the decision-making process is loaded with doubts, the way in which the process unfolds is important for trust in the final decision. A profound understanding of the critically ill patient's clinical situation, as well as the doubts of the referring doctor, emerges in the encounter between the doctors and the patient and/or their family.

Structural analysis

The structural analysis revealed the three themes 'burdensome doubt', 'emerging understanding and 'responding to moral duties' and further nine subthemes (Table 2). An extended Table 2 with more quotes could be found under supplemental files.

For peer review only

Table 2. Structural analysis with themes and subthemes

Themes	Burdensome doubt	Emerging joint understanding	Responding to mutual moral duties
Subthemes	Being affected by the gravity of critical illness	Comprehension by seeing the patient at the bedside	Meeting in person to acknowledge concern
	Pondering about what is meaningful for the patient	Requiring senior experience to grasp clinical complexity	Protecting the patient through advocacy
	Ambivalence in searching for the patient's voice	Needing engagement for broadened perspectives	Building relationships for confidence

For peer review only

Burdensome doubt

The consultants describe a struggle to make the right decision for the critically ill patient under difficult conditions while being highly aware of the risks of death and suffering. The burdensome doubt of the decision-making process signifies a need for trust in the process.

The doctors are affected by the gravity of the critical illness which is imprinted with urgency and precariousness. The patients are perceived as being exhausted and often very affected by their critical condition. This places a moral responsibility on the referring doctor to make the right decision about the further care of the patient.

"It was an extremely difficult decision … For me, admitting her to the intensive care unit and supporting her respiratory system was as much about the symptomatology and control and actually good palliative care rather than … to turn this around. … that the patient's very conscious and gets very breathless, very worked up, very anxious and very frightened." (Haematology consultant, Hospital 2)

Pondering about what is meaningful for the patient refers to considering whether further escalation to intensive care would be good and beneficial for this particular patient. This requires the doctor to obtain a clinical overview of the patient's medical situation in the face of an often rapid change in their condition. The clinical scenario is often experienced as being

For peer review only - http://bmjopen.bmj.com/site/about/guidelines.xhtml

unclear with a lack of information and, by trying to articulate the precise clinical needs of the patient, they could ponder what intensive care could offer the patient.

"I can do lots of technological things but I'm not sure it gives people a good death and I'm not sure it gives people a good quality of life for their last few weeks." (Internal Medicine consultant, Hospital 2)

Doctors experience ambivalence in searching for the patient's voice and involving the patient and family in the decision-making process. They acknowledge on the one hand that the patient and family are natural partners in the process, but, on the other hand, their voice is experienced by the doctors as ambiguous. The doctors reflect on the patient and family's ability to understand the different possibilities for the further care, particularly when the patients are often too sick and exhausted to voice anything at all. They reflect on how patient and family´s wishes for treatment could be driven by fear as well as a lack of realism about what intensive care could achieve.

"We should try and involve the patient, [but] she was clearly not in a position to be asked. We should try and ascertain her current and previous wishes. She was unable to give her current wishes … So we discussed with the family what they think she would have wanted and I think they were unsure as well." (Geriatric consultant, Hospital 2)

Emerging joint understanding

The decision-making process implies multiple perspectives and therefore needs to stimulate an emerging joint understanding of the patient's precarious situation from the perspectives of the different stakeholders. Understanding emerges through comprehending the clinical situation at the bedside, through the eyes of experienced clinicians as well as through engagement with broadened perspectives on the patient's situation. A profound understanding of the critical illness situation is essential for developing trust in decisions about the further care of the patient.

Comprehension by seeing the patient at the bedside means that the bedside encounter with the patient contextualizes the medical information that is available from multiple other sources, such as files, tests and examinations, and, thereby, the unique situation of the particular patient can emerge. Seeing the patient at the bedside means gaining a comprehensive understanding of the patient's situation and the importance of this in person encounter with the patient extends to both the referring doctor and the intensivist.

" First of all, undoubtedly the reality that it's a person, you see them, so if somebody phones me it's a list of numbers, blood pressure, observations I get because I'm a kidney physician. Whereas when you go to see somebody, they're real, they've got family and when you're then thinking about it you contextualize it, you think about this is a real person." (Nephrology consultant, Hospital 3)

Patients could be in more substantial need of intensive care than the numerical physiological measures and telephone reports have suggested. Initial reluctance from the intensivist to admit

For peer review only - http://bmjopen.bmj.com/site/about/guidelines.xhtml

a patient can rapidly change when they see the patient. When intensivists dismiss the need for admission to intensive care without seeing the patients, the referring doctors regard this as irresponsible practice.

Senior clinical experience at a consultant level is required to be able to grasp the clinical complexity and to understand critical illness as a dynamic condition. Being able to anticipate the unpredictability of the situation and make anticipatory plans for further care of the patient helps to secure the safety of the patient as well as the doctor's own confidence in the unpredictable situation.

"Especially when it's the patients that could get a lot worse, it's almost a seniority thing. They [junior doctors] don't know enough, haven't seen enough to realize that the information that you're imparting is because we're basing it on experience of things going worse." (Acute and Emergency Medicine consultant, Hospital 1)

Needing engagement for broadened perspectives refers to the mutual responsibility of both the referring doctor and the intensivist to actively seek more information about the patient's situation in order to broaden their own perspectives and contribute to a more profound understanding of the patient's situation.

Referring doctors describe their need to obtain the perspective from intensive care on what can be done as well as a realistic view on what it is possible to do for a critically ill patient. They describe how they experience only being able to judge the most acute needs of the patient and acknowledge the clinical situation over a short period of time and that the intensivist's judgment contributes a broader perspective by providing new insights and understanding. The intensivist is experienced as an important sounding board for the referring doctor.

"You're not quite sure, you just know someone's ill and you're just trying to work out what is it that I need and then that consultant who I spoke to is very good at sort of saying: 'Well what is it you're actually asking for? Let's work this out!'" (Internal Medicine consultant, Hospital 1)

However, experiences of lack of engagement from the intensivist and a too-narrow perspective on the patient's situation means a lack of understanding of the patient's difficult situation and an increased burden on the referral doctor with loss of trust.

"That's a very easy thing to do, to walk in and say: 'Not fit for intensive care,' then wander back to your intensive care unit. That's quite an easy decision to make. You're not then the person who's there, dealing with that patient, that family saying: 'Why aren't you treating my mum?'" (Colorectal surgeon, Hospital 5)

Responding to mutual moral duties

Fulfilling moral duties is part of the process of gaining trust in the decision-making process and this is achieved in the encounter between the doctors.

For peer review only - http://bmjopen.bmj.com/site/about/guidelines.xhtml

Meeting in person to acknowledge concern refers to the need to meet face-to-face with the intensivist. Meeting in person facilitates discussion, new questions are answered directly, and it is easier to make further plans for the patient's care. The referring doctor's concern for the critically ill patient can be acknowledged and more confidence in the decision can be gained by sharing the burden of making such a complex decision.

"People who come to see the patient, make a proper assessment, talk to me and explain to me, I can ask questions and we come to an agreement. I think that's straightforward. There the trust grows very quickly." (Respiratory physician, Hospital 6)

The referring doctors see themselves as having a duty to advocate on behalf of the patient when there is disagreement and lack of confidence about the further care of the patient. This includes arguing for the possible benefit of intensive care. Advocacy is seen as being important when bed capacity is a concern. The referring doctors are aware of the intensivists' concern of capacity. However, they see that their obligation is to strictly focus on the needs of their critically ill patient:

"Beds are tough but, that's not a part of the decision-making; is the patient going to get better care on ICU than where they are now? Can we do something to intervene and then if the decision-making is 'yes' and if appropriate for the patient then the final [a bed] is secondary." (Haematology consultant, Hospital 6)

Advocacy also involves arguing for the time needed in ICU to get a clearer picture of the medical condition. Admission with a restriction in length of stay or type of intervention could be offered to give the patient a chance, but at the same time restricts long-term suffering. These negotiations are a way of operationalizing the duty of protection from harm.

Building relationships between the referring doctor and the intensivist facilitates confidence and further trust in the decision. This is achieved through learning to know each other while sharing the care for patients. Although the intensivist makes the formal decision about an admission, the referring doctors describe their own duties in making discerning and adequate clinical judgements to contribute to a necessary and mutual confidence in the process.

A strong, positive relationship could mean that the referring doctors ignore the formal referral system and directly contact senior intensive care colleagues to discuss their patients. The referring doctors also describe having a personal and extended responsibility for patients being cared for on the intensive care unit, particularly for patients who they have known for a long time, but also patients suffering from complicated or rare diseases, where they regard their knowledge and experience to be important. This responsibility is grounded in a personal relationship of mutual confidence and support that the referring doctors are eager to foster.

"I have a good relationship with the more senior ICU consultants and I suppose it all is dependent on trust. I trust their judgement and they trust my judgement and that is based on years of working with each other and seeing what we do and how we make decisions ... it comes from experience, so if I referred patients to the intensive care unit and they took them

For peer review only - http://bmjopen.bmj.com/site/about/guidelines.xhtml

and then felt time and time again they weren't appropriate referrals they would stop trusting my judgement." (Nephrology consultant, Hospital 3)

Comprehensive understanding

The structural analysis of referring doctors' experience of the decision-making process revealed the burdensome doubt of the referring doctor, how to contribute to an emerging, joint understanding of the patient´s situation and mutual moral duties that need response. Previous research has shown tension and misunderstandings and the need for improved communication between ICU and ward teams (11, 13, 36). Our study shows from the perspective of referring consultants, how trust in the decision-making process could be enhanced through the way the process unfolds in the relations between the patient and the stake holding doctors. When the clinical scenario does not have an obvious right or wrong answer to what ought to be done, *the way* in which the decision is made and *how* the process unfolds, is important for gaining trust in the decision-making process. The further comprehensive understanding of the data was developed in light of the writings of Emmanuel Levinas and the concept of 'Ethics of proximity'(33, 34).

The face-to-face encounter, and the moral responsibility that manifests itself in this encounter, is central to Levinas' philosophy (37). It deals with the existential character of human relations and signifies a search for deeper patterns of being human in relation to others. Levinas describes how, in the encounter with 'The Other's Face', physical presence is not foremost; rather, we are called by 'The Other's Face' and responding to that 'otherness'. The Other is substantially different from me and this otherness establishes a basic asymmetry in the relation between us. Based on the asymmetrical relation of otherness as well as proximity when encountering each other´s faces, comes the moral claim of protection and responsibility.

Seeing the critically ill patient in person bedside is important in the decision-making process and is true for both the referring doctor as well as the intensivist. This gestalt assessment of the patient has previously been shown to be important in the decision-making process (14). Seeing the patient could be further interpreted, in light of Levinas' philosophy, as being an expression of the demand to encounter the critically ill patient as The Other's Face, and so recognize, through a clinical proximity, the moral claim for responsibility in a critical and vulnerable situation. Seeing the face of the patient adds substantial moral information to the process – what is at stake and what ought to be done – over and above the medical information. In the asymmetrical relation with The Other, we encounter, according to Levinas, The Other's, as well as our own, vulnerability, and the empirical findings reveal what could be interpreted as a substantial vulnerability (34). The critically ill patient is perceived as being vulnerable, imprinting gravity in the decision-making process. The referring doctor's burdensome doubt exposes their own vulnerability – being close to the existential dimensions of life and burdened by the seriousness and uncertainty of the decision.

Levinas describes how, in the encounter with The Other's Face, we are called to protect and take responsibility. This is in line with the referring doctors' advocacy for the patient when they think the patient should be admitted to the ICU and could be seen as a clinical outlook of protection and responsibility. However, the moral claim of responsibility and protection will

For peer review only - http://bmjopen.bmj.com/site/about/guidelines.xhtml

For peer review only

not in itself inform which decision to make or what is the right thing to do, but could be seen as an underlying prerequisite for making a moral judgement, through clinical and moral proximity, with the patient as well as with the intensivist. When the proximity in experienced as responsible by the referring doctors as highlighted in the structural analysis, trust in the decision-making process could be enhanced.

Proximity in ethics has been described as an important part of moral perception. Moral perception is used to describe our capacity to acknowledge morally salient aspects of a particular situation (38). Moral perception, to see that something matters morally, can be distinguished from moral reasoning, a more conscious deliberation about action and reasons for actions (39). In the present study, the importance of clinical proximity could also be interpreted as being important for the moral perception of a critical situation. Being close to the patient, the moral obligation of the doctors to always see the patient at the bedside, could contribute to identifying morally salient aspects of the patient's situation that are not detectable over the phone or in notes and could be seen as a component of enhancing trust in the decision-making process.

The findings contribute to an increased understanding of the ethical as well as clinical basis for the known importance of relationships between clinicians and their patients in the complex decision-making process of referral to intensive care. This model of the interaction between the intensive care and referring clinical teams is characterised by doubt, resolved through discussion to a shared understanding and assuming of moral responsibility, and from that trust in the decision-making process could emerge.

The moral understanding that this analysis provides supplements the clinical understanding by identifying important duties, trusts and relationships. When these duties and relationships breakdown it is likely to negatively impact the patients who rely on the care that can only be provided as part of a broad hospital team as well as negatively impact working relationships.

METHODOLOGICAL CONSIDERATIONS

The data were not collected with the intention of using a phenomenological hermeneutic analysis method, which could pose a potential threat to validity. However, we regarded the data as being rich with the possibility of capturing meanings of lived experiences. The first author, who led the analysis, did not conduct any interviews, which could be seen as a weakness. However, the data analysis process was conducted under constant critical review and reflection by the co-authors with different kinds of preunderstandings. The first author (KH) has clinical experience as a referring medical doctor and the rest have clinical experience from general practice (AS, FG), intensive care nursing (MS) and ambulance care (AB). Three were involved in the data-collection and the rest of the project (MS, AS, FG) and coming from outside (AB), ensuring trustworthiness.

It can be argued that the sample size was too large for this method, thus challenging the depth of the analysis when dealing with the large quantity of data. But it may also be regarded as a

For peer review only - http://bmjopen.bmj.com/site/about/guidelines.xhtml

strength, as we captured varied narratives from different clinical specialties as well as types of hospital.

The clinical context of the study is the UK, but the findings might be transferable to similar organisations of healthcare in developed countries. However, this is in the eye of the reader to judge.

CONCLUSION

This study has provided an increased understanding of the interaction between the moral and clinical aspects around referral to intensive care. The paper has articulated a rationale for the important joint assessment of a patient at the bedside that is well recognised but often neglected.

The findings highlight the importance of trust in the decision-making process and how the relationships between the stake-holding doctors are crucial to support their moral responsibility for the patient. Poor interpersonal relationships can damage trust and negatively impact decisions made on behalf of a critically ill patient. For this reason, active attempts must be made to foster good relationships between doctors. This is not only important to create a positive working environment, but a mechanism to improve patient outcomes.

The study has clinical implications for guidance to support the process of referral to intensive care and should be used to improve the referral process. Hospitals should consider specifying best practice in referrals, including joint bedside assessment, as described in the wider ICU referral and admission decision-making project (11).

Author statement

Kaja Heidenreich: Consultant internal medicine and cardiology, PhD student, conducted the coding, led the analysis and wrote the manuscript.

Anne Slowther: Main project leader, clinical ethicist, conducted two interviews, scrutinized the analysis and reviewed the manuscript.

Frances Griffiths: Leader of the project work-package in which data were collected, UK general practitioner and sociologist, conducted six interviews and reviewed the manuscript.

Anders Bremer: Ethics researcher, ambulance nurse and specific expertise in phenomenological-hermeneutical analysis, scrutinized this analysis, and reviewed the manuscript.

Mia Svantesson: Ethics researcher, ICU nurse and expertise in qualitative methods, conducted 19 interviews, scrutinized the analysis and reviewed the manuscript.

For peer review only - http://bmjopen.bmj.com/site/about/guidelines.xhtml

Competing Interest

There are no competing interests for any author.

Acknowledgment

We are grateful to the consultants who participated in the study.

Dr Chris Bassford was Chief Investigator for the larger project looking at decision making around admission to intensive care in which the data analysed in this paper were collected. Dr Bassford has provided helpful comments on the revised manuscript to make it more readable for clinicians and suggested revisions to the conclusion to highlight the clinical implications.

Data availability

No additional data available.

Funding

This article presents independent research funded by the National Institute for Health Research (NIHR) under the Health Services and Delivery Research programme (project number 13/10/14).

Disclaimer

The views expressed in this publication are those of the authors and not necessarily those of the NIHR or the Department of Health and Social Care.

For peer review only - http://bmjopen.bmj.com/site/about/guidelines.xhtml

REFERENCES

1. Brinkman S, de Jonge E, Abu-Hanna A, Arbous MS, de Lange DW, de Keizer NF. Mortality after hospital discharge in ICU patients. Crit Care Med. 2013;41(5):1229-36.
2. Williams TA, Dobb GJ, Finn JC, Knuiman MW, Geelhoed E, Lee KY, et al. Determinants of long-term survival after intensive care. Crit Care Med. 2008;36(5):1523-30.
3. Montuclard L, Garrouste-Orgeas M, Timsit JF, Misset B, De Jonghe B, Carlet J. Outcome, functional autonomy, and quality of life of elderly patients with a long-term intensive care unit stay. Crit Care Med. 2000;28(10):3389-95.
4. ICNARC. Key statistics from the Case Mix Programme - Adult, general critical care units 1, April 2015 to 31 March 2016. 2017.
5. Mnatzaganian G, Galai N, Sprung CL, Zitser-Gurevich Y, Mandel M, Ben-Hur D, et al. Increased risk of bloodstream and urinary infections in intensive care unit (ICU) patients compared with patients fitting ICU admission criteria treated in regular wards. J Hosp Infect. 2005;59(4):331-42.
6. Azoulay E, Pochard F, Kentish-Barnes N, Chevret S, Aboab J, Adrie C, et al. Risk of post-traumatic stress symptoms in family members of intensive care unit patients. Am J Respir Crit Care Med. 2005;171(9):987-94.
7. Garrouste-Orgeas M, Perier A, Mouricou P, Gregoire C, Bruel C, Brochon S, et al. Writing in and reading ICU diaries: qualitative study of families' experience in the ICU. PLoS One. 2014;9(10):e110146.
8. Wade DM, Howell DC, Weinman JA, Hardy RJ, Mythen MG, Brewin CR, et al. Investigating risk factors for psychological morbidity three months after intensive care: a prospective cohort study. Crit Care. 2012;16(5):R192.
9. Storli SL, Lindseth A, Asplund K. A journey in quest of meaning: a hermeneutic-phenomenological study on living with memories from intensive care. Nurs Crit Care. 2008;13(2):86-96.
10. Svenningsen H, Egerod I, Dreyer P. Strange and scary memories of the intensive care unit: a qualitative, longitudinal study inspired by Ricoeur's interpretation theory. J Clin Nurs. 2016;25(19-20):2807-15.
11. Bassford C, Griffiths F, Svantesson M, Ryan M, Krucien N, Dale J, et al. Gatekeeping in intensive care: Understanding and improving the decision-making process surrounding admission to the intensive care unit. University Hospitals Coventry & Warwickshire NHS Trust; 2018.
12. Griffiths F, Svantesson M, Bassford C, Dale J, Blake C, McCreedy A, et al. Decision-making around admission to intensive care in the UK pre-COVID-19: a multicentre ethnographic study. Anaesthesia. 2020.
13. Svantesson M, Griffiths F, White C, Bassford C, Slowther A. Ethical conflicts during the process of deciding about ICU admission: an empirically driven ethical analysis. J Med Ethics. 2021.
14. Bassford CR, Krucien N, Ryan M, Griffiths FE, Svantesson M, Fritz Z, et al. U.K. Intensivists' Preferences for Patient Admission to ICU: Evidence From a Choice Experiment. Crit Care Med. 2019;47(11):1522-30.
15. Uy J, White DB, Mohan D, Arnold RM, Barnato AE. Physicians' decision-making roles for an acutely unstable critically and terminally ill patient. Crit Care Med. 2013;41(6):1511-7.
16. McNarry AF, Goldhill DR. Intensive care admission decisions for a patient with limited survival prospects: a questionnaire and database analysis. Intensive Care Med. 2004;30(2):325-30.
17. Einav S, Soudry E, Levin PD, Grunfeld GB, Sprung CL. Intensive care physicians' attitudes concerning distribution of intensive care resources. A comparison of Israeli, North American and European cohorts. Intensive Care Med. 2004;30(6):1140-3.
18. Garrouste-Orgeas M, Tabah A, Vesin A, Philippart F, Kpodji A, Bruel C, et al. The ETHICA study (part II): simulation study of determinants and variability of ICU physician decisions in patients aged 80 or over. Intensive Care Med. 2013;39(9):1574-83.

For peer review only - http://bmjopen.bmj.com/site/about/guidelines.xhtml

19. Cooper AB, Joglekar AS, Gibson J, Swota AH, Martin DK. Communication of bed allocation decisions in a critical care unit and accountability for reasonableness. BMC Health Serv Res. 2005;5:67.
20. Mielke J, Martin DK, Singer PA. Priority setting in a hospital critical care unit: qualitative case study. Crit Care Med. 2003;31(12):2764-8.
21. Giannini A, Consonni D. Physicians' perceptions and attitudes regarding inappropriate admissions and resource allocation in the intensive care setting. Br J Anaesth. 2006;96(1):57-62.
22. Blanch L, Abillama FF, Amin P, Christian M, Joynt GM, Myburgh J, et al. Triage decisions for ICU admission: Report from the Task Force of the World Federation of Societies of Intensive and Critical Care Medicine. J Crit Care. 2016;36:301-5.
23. Young PJ, Arnold R. Intensive care triage in Australia and New Zealand. N Z Med J. 2010;123(1316):33-46.
24. Pintado MC, Villa P, Gonzalez-Garcia N, Lujan J, Molina R, Trascasa M, et al. Characteristics and outcomes of elderly patients refused to ICU. TheScientificWorldJournal. 2013;2013:590837.
25. Iapichino G, Corbella D, Minelli C, Mills GH, Artigas A, Edbooke DL, et al. Reasons for refusal of admission to intensive care and impact on mortality. Intensive Care Med. 2010;36(10):1772-9.
26. James FR, Power N, Laha S. Decision-making in intensive care medicine - A review. J Intensive Care Soc. 2018;19(3):247-58.
27. Rees S, Bassford C, Dale J, Fritz Z, Griffiths F, Parsons H, et al. Implementing an intervention to improve decision making around referral and admission to intensive care: Results of feasibility testing in three NHS hospitals. J Eval Clin Pract. 2020;26(1):56-65.
28. Oerlemans AJ, van Sluisveld N, van Leeuwen ES, Wollersheim H, Dekkers WJ, Zegers M. Ethical problems in intensive care unit admission and discharge decisions: a qualitative study among physicians and nurses in the Netherlands. BMC Med Ethics. 2015;16:9.
29. Halvorsen K, Forde R, Nortvedt P. Value choices and considerations when limiting intensive care treatment: a qualitative study. Acta Anaesthesiol Scand. 2009;53(1):10-7.
30. Nates JL, Nunnally M, Kleinpell R, Blosser S, Goldner J, Birriel B, et al. ICU Admission, Discharge, and Triage Guidelines: A Framework to Enhance Clinical Operations, Development of Institutional Policies, and Further Research. Crit Care Med. 2016;44(8):1553-602.
31. Lindseth A, Norberg A. A phenomenological hermeneutical method for researching lived experience. Scand J Caring Sci. 2004;18(2):145-53.
32. Ricoeur P. Interpretation theory, discourse and the surplus of meaning. Fort Worth, Texas: TCU Press; 1976.
33. Levinas E. Totality and Infinity. An Essay on Exteriority. Pittsburgh: Duquesne University Press; 1969.
34. Bergo B. Emmanuel Levinas. In: Zalta E, editor. Stanford Encyclopedia of Philosophy. Fall 2017 Edition ed. Stanford: The Metaphysics Research Lab, Center for the Study of Language and Information, Stanford University; 2017.
35. O'Brien BC, Harris IB, Beckman TJ, Reed DA, Cook DA. Standards for reporting qualitative research: a synthesis of recommendations. Acad Med. 2014;89(9):1245-51.
36. Rees S, Griffiths F, Bassford C, Brooke M, Fritz Z, Huang H, et al. The experiences of health care professionals, patients, and families of the process of referral and admission to intensive care: A systematic literature review. J Intensive Care Soc. 2020;21(1):79-86.
37. Levinas E. Ethics and Infinity. Conversations With Philippe Nemo. Pittsburgh: Duquesne University Press; 1985.
38. Blum L. Moral perception and particularity. New York: Cambridge University Press; 1994.
39. Richardson HS. Moral Reasoning. In: Zalta EN, editor. The Stanford Encyclopedia of Philosophy. Winter 2014 ed: The Metaphysics Research Lab, Center for the Study of Language and Information, Stanford University; 2014.

For peer review only - http://bmjopen.bmj.com/site/about/guidelines.xhtml

Themes	Subthemes	Quotes
Burdensome doubt	Being affected by the gravity of critical illness	*…and just conveyed to them (intensive care team) of how unwell this patient was and if there was a window of opportunity that it was only going to be a very small one.* (Acute and Emergency Medicine consultant, Hospital 1)<br><br>*It's a life or death situation that needs to have a thorough discussion.* (Renal and Emergency Medicine consultant, Hospital 6)<br><br>*It's that reassurance that it has been taken seriously and it's not a light decision. People have perhaps thought about you know the alternative to intensive care. So I think it's very easy to say, "Well this person's not for intensive care," without thinking what, what does that mean for the patient? What are you going to do instead?* (Internal Medicine consultant, Hospital 2)
	Pondering about what is meaningful for the patient	*The chances of there being a worsening function are high. So very often we might get somebody to survive but they won't walk.* (Geriatric consultant, Hospital 2)<br><br>*But I don't think ultimately he would benefit from it, I think the burden of it would probably outweigh any likelihood and the best you could hope for would be to get him back to his pre-morbid condition which was happy and smiley, interacting a little but completely physically dependent. So I'm not sure that would be a helpful thing for him.* (Internal Medicine consultant, Hospital 2)<br><br>*When I feel that it wouldn't be beneficial for them to go to intensive care and that's sometimes the patients that are very frail or have a lot of comorbidities who in my experience of intensive care, my interaction with them, I don't think would be successful, I don't think ICU would change their end kind of outcome, where I feel we would be doing more harm to them that the benefit.* (Acute and Emergency Medicine consultant, Hospital 1)
	Ambivalence in searching for the patient's voice	*Well again I think we try and involve the family but often again although they're in a slightly better position in that they're not ill when they're trying to weigh up decisions, it's still difficult to grasp all the information and the implications of it.* (Renal and Emergency Medicine consultant, Hospital 5)<br><br>*A patient who's clearly just reaching the end of their life, becomes desperate and wants you to try everything. … we're inviting the patients to give their opinion but then we're going to ignore it if we think it's the wrong thing,*

For peer review only - http://bmjopen.bmj.com/site/about/guidelines.xhtml

		that's very difficult for medicine to deal with them. I don't know how we should approach that really. (Cardiology consultant, Hospital 5)<br><br>*Not to lose sight of what we think is best for the patient and to make sure that we stick to the factors that present themselves and that we make level headed decisions for the patients and I think we are very aware that patients may have got an overly optimistic view of what ICU can achieve.* (Oncology consultant, Hospital 2)
Emerging joint understanding	Comprehension by seeing the patient at the bedside	*There are some patients as I say in that grey area that I feel very much it's eyeballing the patient and getting a feel for them as to what that decision should be and in those patients I think that your personal review is important. Just like when I make a decision as to whether somebodies going to go to theatre or not.* (Colorectal surgeon, Hospital 5)<br><br>*If they're starting to get tired, they look tired, you can sometimes by looking at them sort of, sometimes it's a gut feeling. I know that is difficult to put in a guideline isn't it?* (Neurology consultant, Hospital 6)<br><br>*You find a very different picture and the patient's much weller than you expected them to be or much sicker than you expected them to be. Y it's hard to say, "No," when you've seen the patient, you have to justify that and so you have to be more of a doctor.* (Acute and Emergency Medicine consultant, Hospital 4)
	Requiring senior experience to grasp clinical complexity	*Sometimes things may happen too acutely and maybe out of hours for that initial consultant to consultant discussion to happen but I think in a, in a sort of fairly short time frame there ought really to be a consultant and consultant discussion about the patient so that both sides can fully understand the context.* (Haematology consultant, Hospital 3)<br><br>*At the end of the day there's going to be no substitute for a trained clinician, who's experienced to handle these quite complex sort of pictures and to guide through those sort of decisions.* (Infectious Disease consultant Hospital 5)<br><br>*Trying to do it in a thoughtful senior clinician based manner rather than it's 3 in morning what should we do?* **(**Internal Medicine consultant, Hospital 2)
	Needing engagement for broadened perspectives	*Who (intensive care team) are extremely helpful and are always happy to listen and always happy to come and see the patient and have a positive, you know, constructive discussion.* (Oncology consultant, Hospital 2)

For peer review only - http://bmjopen.bmj.com/site/about/guidelines.xhtml

		They're coming from a different angle, I think usually if you phrase this on not sure what the best solution is here because of x, y, z and then talk that through that does tend to be the better sort of collaborative sort of approach. (Acute and Emergency Medicine consultant, Hospital 1)<br><br>*There are situations where I'm very confident that I know with that clinical situation well enough to be able to make very clear decisions, I've got no hesitation of doing that. But there are situations where there is genuine doubt and the view would be at that stage having somebody else from a different, coming from a different angle, who's very helpful to see and if critical care felt that they were able to do something to help them then that would be fine* (Hepatology consultant, Hospital 2)
Responding to mutual moral duties	Meeting in person to acknowledge concern	*It's just different, you can do it on the phone but you don't get the same, on a phone it's very hard to relay all that information, we've all been on call and at the end of the phone and you just don't get a picture of what's going on, whereas when you talk to somebody you ask different questions, you have a much, it's a more informal discussion I think when it's face to face.* (Colorectal surgeon, Hospital 5)<br><br>*So I think that, that communication is absolutely crucial and sometimes you know there's something that you may not have thought about but, you actually know the answer to and, rather than this going backwards and forwards if you have either a direct discussion or you're present at the time the discussion is being had, I find that very helpful.* (Oncology consultant, Hospital 2)<br><br>*So it's about a conversation between 2 clinicians and I, I honestly still believe the best, that's best on face to face where ever possible, it's not always possible but where ever possible.* (Colorectal surgeon, Hospital 5)
	Protecting the patient through advocacy	*When I'm referring a patient to ITU, I am acting as that patient's advocate and THAT patient's advocate only and the capacity is somebody else's problem. By heck! That is my patient and I want the best for that patient!* (Respiratory physician consultant, Hospital 6)<br><br>*I see myself as the advocate of the patient because he's on my ward, so I stand for him and ITU needs to assess and then needs to convince me why not. That is really my take. Because I would not ask if I don't want the patient to lie in intensive care.* (Renal and Emergency Medicine consultant, Hospital 6)<br><br>*Whether you have a bed or not, I'm not asking you if you've got a bed, I'm asking you for your opinion as an intensivist whether you think this patient's condition would best be managed on an intensive care unit. Once we've decided that*

For peer review only - http://bmjopen.bmj.com/site/about/guidelines.xhtml

		is the best place we can then decide where we can find an intensive care bed or how we could facilitate a bed being available. (Renal Medicine consultant, Hospital 3)
	Building relationships for confidence	*If they know you and they trust you and they know what, what you usually do or whether you look after your patients and how you work, most of you will then take that on board and say, "Well fine…"* (Colorectal surgeon, Hospital 5)<br><br>*If I go to them and say, "Look this is the difficulty I have," I've always found, I've always found everybody to be quite reasonable and try to accommodate and weighing up the risks and benefits of the patient. I've never found an ITU consultant to be defensive and saying they don't want to take a patient or to block a patient off, that's not really been the case. If you can, if they can understand why you're doing something and what the surgery's about but that's also I think… the factor of them whether they learn that what you do is reasonable or not. I think it depends on your relationship with them and some cases I think perhaps there will be a lesser understanding for why somebody wants a patient on ITU if historically that decision making has not always been good form the surgeon's side.* (Orthopedic surgeon, Hospital 2)<br><br>*…they know that we don't ask for flippant requests and that if we want help, we need it and so I think that they're extremely supportive.* (Haematology consultant, Hospital 1)

For peer review only - http://bmjopen.bmj.com/site/about/guidelines.xhtml

THE UNIVERSITY OF WARWICK
WARWICK MEDICAL SCHOOL

REC number: 15/WM/0025

Understanding and improving the decision-making process for treating people who are critically ill

Non referring clinicians

1. I am just curious to know how come you volunteered for this interview?

2. In the past three weeks have you referred to ICU any patients under your care?

   A) **If yes,** starting with the most recent patient, can you talk me through how you made the decision to contact the critical care team (repeat for up to 5 cases)
   Follow-up: Who did you contact and for what reason? What do you think influenced you when making this decision? Circumstances? Persons? What was the outcome, admission to ICU or not? What do you think was the most important to take into account when you made the decision?

   B) **If no**, did you consider referring patients, but made the decision not to? Please, tell me about how you reasoned about not referring?
   Follow-up: *What do you think influenced you when making this decision? Circumstances? Persons? What do you think was the most important to take into account when you made the decision?*

   C) **If you have not considered referral**, can you talk me through reasons for not referring to ICU?

3. What is your general experience of the decision-making referring patients to ICU? *Follow-up: How do you think your way of making decisions to refer compare with your colleagues?*

4. Thinking more generally, how could decision making whether to admit patient or not to ICU be improved?

5. What type of support would be useful when facing this type of decision? *Follow-up: What format of this support would be useful?*

6. Are you aware of any policies about this kind of decision-making?

Background
Age:
Specialty:
Years as consultant:
Graduation year:

IS3 clinician interviews WP1 V6.1 23.10.15

For peer review only

Reporting checklist for qualitative study based on the SRQR guidelines

Title and abstract

Item 1 See Title page

Item 2 See Abstract

Problem formulation

Item 3 See "Background" page 3.

Purpose or research question

Item 4 See "Background" page 3.

Qualitative approach and research paradigm

Item 5 See "Design" page 4

Researcher characteristics and reflexivity

Item 6 See "Author statement" page 14

Context

Item 7 See "Participants and setting" page 4

Sampling strategy

Item 8 See "Participants and setting" page 4

Ethical issues pertaining to human subjects

Item 9 See "Ethical considerations" page 5

Data collection method

Item 10 See "Data collection" page 5

Data collection instruments and technologies

Item 11 See "Data collection" page 5

Units of study

Item 12 See "Participants and setting" page 4 and Table 1

Data processing

Item 13 See "Data analysis" page 5-6

For peer review only

Data analysis

Item 14 See “Data analysis” page 5-6

Techniques to enhance trustworthiness

Item 15 See “Data analysis” page 5-6

Syntheses and interpretation

Item 16 See “Comprehensive understanding” page 10-12

Links to empirical data

Item 17 Quotes are embedded in the result section to illustrate the findings.

Integration with prior work, implications, transferability and contribution(s) to the field

Item 18 See “Comprehensive understanding” page 10-12

Limitations

Item 19 See “Strengths and limitations” on page 1

Conflict of interest

Item 20 See page 2

Funding

Item 21 See page 14

For peer review only
